# Prognosis following a diagnosis of heart failure and the role of primary care: a review of the literature

**DOI:** 10.3399/bjgpopen17X101013

**Published:** 2017-10-04

**Authors:** Nicholas R Jones, FD Richard Hobbs, Clare J Taylor

**Affiliations:** 1 GP Trainee & Academic Clinical Fellow, Nuffield Department of Primary Care Health Sciences, University of Oxford, Oxford, UK; 2 GP & Professor of Primary Care, Nuffield Department of Primary Care Health Sciences, University of Oxford, Oxford, UK; 3 GP & NIHR Academic Clinical Lecturer, Nuffield Department of Primary Care Health Sciences, University of Oxford, Oxford, UK

**Keywords:** heart failure, prognosis, primary care

Heart failure (HF) is a common chronic condition that affects around 900 000 people in the UK.^1^ Primary care plays a central role in the diagnosis, long-term management, and end-of-life care for these patients. While there is specialist support available from HF nurses and cardiologists, GPs remain responsible for overseeing most patient care once a diagnosis is made including management to delay progression, recognition of HF decompensations, and patient follow-up in the vulnerable period following an acute admission. Patients with HF often have several other conditions, which can change over time and require different, sometimes conflicting, treatment. GPs also provide timely information on prognosis, discuss treatment options, and support advanced care planning.^2^ It is therefore crucial to support clinicians’ understanding and awareness of the HF trajectory. In this article we present relevant evidence on HF survival rates and factors that affect outlook. We also explore reported cause of death in patients with HF and trends in survival over time.

## Acute and chronic heart failure

The distinction between acute and chronic HF may be artificial, as both usually occur at some point in most patients with HF but clinical practice and research have traditionally separated the two groups. When discussing prognosis with a patient it can be useful to consider their recent level of symptom stability as the evidence base draws on studies that have tended to recruit from either stable, ‘chronic’ community patients or those admitted with an ‘acute’ episode of HF. The European Society of Cardiology's (ESC's) definition of chronic HF is where the patient has had the condition for some time; stable is where there has been no significant change in the past month.^3^ A chronic, stable patient who suffers a deterioration in their condition can be said to have acutely decompensated, but ‘acute HF’ can also refer to a patient’s first presentation with HF.^1^

There is a growing recognition that these delineations are somewhat arbitrary and there is in reality a spectrum of fluctuant symptom stability on a background of clinical deterioration over time. Many patients, following their initial diagnosis, may enjoy a period of relatively prolonged stability, sometimes providing false reassurance that HF is a benign or easily managed condition. These periods of stability often become interrupted by increasingly frequent decompensations, when poor symptom control may require a step-up in the intensity of treatment and frequent acute episodes of HF can be seen as a poor prognostic sign. There are often opportunities for GPs to initiate early intervention in these situations to reduce the severity of acute episodes and potentially to avoid admissions by, for example, involving community services that provide home or near home treatment. For instance, in some areas community teams are able to initiate potent diuretic combinations, such as the addition of metolazone to furosemide, or consider closely monitored parenteral treatments where gastrointestinal absorption is compromised due to fluid overload.^4^

A number of large, international cohort studies have looked at prognosis in patients with chronic HF, either managed in the community or outpatient setting, and report a 1-year survival rate of around 80–90%, compared with 97% in the general population (Table 1).^5–11^ There is similar concordance at 5 years with survival around 50–60% compared with 85% in the general population.^5–7^ Ten-year survival for patients with HF and left ventricular systolic dysfunction (LVSD) in the UK community-based Electrocardiographic Heart of England (ECHOES) study was 27.4%, compared with 75% for the general population.^10^ These figures are supported by a recent survival analysis from UK primary care data of 54 313 patients with a first diagnosis of HF, showing survival rates of 81.3%, 51.5%, and 29.5% at 1, 5 and 10 years respectively.^11^

**Table 1.** Survival rates in chronic heart failure

Study	Setting	Total participants	Participants with HF	Country	Average age at outset, years	1-year survival, %	2-year survival, %	5-year survival, %	10-year survival, %
Mosterd *et al* 2001^5^	Participants from Rotterdam population study screened for diagnosis or symptoms of HF	5255	181	The Nether-lands	68.9	89	79	59	–
Nielsen* et al* 2003^6^	Cross-sectional sample from three general practices, screened for participants with diagnosis or symptoms of HF	2157 including control group (571) and heart disease but no HF (218)	67 with community managed HF: 33 in hospital managed HF comparator	Denmark	74.1	–	–	61	–
Hobbs *et al* 2007^7^	Participants from UK general practice screened for HF	6162 including 1062 at high risk for HF and 982 on diuretics at baseline	Previous clinical label of HF ( 782)	UK	64.2	–	–	63	–
Tsutsui *et al* 2007^8^	Data from The Cardiac Registry of Heart Failure in General Practice	2685	1280 followed-up in hospital and 1405 by GP	Japan	74	90.9	–	–	–
Pons* et al* 2010^9^	Consecutive sample from outpatient HF clinic	960	All followed-up in outpatient cardiology clinic	Spain	69	89.4	–	–	–
Taylor *et al* 2012^10^	As per Hobbs *et al* 2007 paper:^7^ 10-year follow-up of ECHOES cohort	See above	See above	UK	64.2	–	–	–	26.7
Taylor *et al* 2017^11^	Survival analysis using UK primary care records from The Health Improvement Network	54 313﻿	All with first diagnosis of HF	UK	76.5	81.3	–	51.5	29.5

ECHOES = Echocardiographic Heart of England Screening (ECHOES). HF = heart failure.

Studies following hospital admission with acute HF have similar concordance for 30-day post-discharge survival; around 80% (Table 2).^6,12–16^ The figure does not include those who die in hospital and the results support the theory that increasing symptomatic relapses are a marker of HF progression. This highlights the 30 days following an acute admission as a high-risk period, and the need for close follow-up and monitoring, including better patient and carer awareness of signs of deterioration (decompensation), timely and informative correspondence from hospitals, and liaison with specialist community teams where available. Longer-term survival outcomes following an acute admission are more heterogeneous, perhaps reflecting the pattern of recurrent symptomatic relapses with periods of stability of variable and unpredictable duration in between. Patients with acute HF tend to be older with a greater proportion being female. Clinical characteristics, including comorbidities and severity of HF at time of diagnosis, are also important in predicting survival.

**Table 2.** Survival rates in acute heart failure

Study	Participants	Total participants	Country	Average age, years	30 day survival, %	1-year survival, %	3-year survival, %	5-year survival, %	6-year survival, %
Blackledge *et al* 2003^12^	Following admission to hospital with first episode of HF	12 220	UK	76.8	79.1	57.1	38.9	27	23.2
Nielsen *et al* 2003^6^	Cross-sectional sample from three general practices, screened for participants with diagnosis or symptoms of HF	2157: 33 in hospital HF group; 67 in GP-managed HF; control group (571) and heart disease but no HF (218)	Denmark	74.1	–	–	–	39	–
Goldberg *et al* 2007^13^	Recruited from 11 sites following admission with acute HF	2445	US	76	–	62.7	–	21.5	–
Ko *et al* 2008^14^	Recruited following first admission with HF	9943	Canada	75.8	–	66.9	–	31.3	–
Jhund *et al* 2009^15^	Every admission with first episode of HF in Scotland during study period	116 556	Scotland	70.7	80.8	55.8	–	26	–
Parenica* et al* 2013^16^	Recruited from seven sites following admission with acute HF	4153	Czech Republic	72.8	82.8	79.7	64.5	–	–

HF = heart failure.

## Preserved and reduced ejection fraction

A diagnosis of HF requires the patient to have symptoms plus objective evidence of a structural or functional abnormality of the heart. The left ventricular ejection fraction (EF) is used to distinguish between HF with reduced EF (HFrEF) and HF with preserved EF (HFpEF). A third category with mid-range EF (HFmrEF) has recently been added to the classification by the ESC (Box 1). This categorisation is important in determining likely response to treatment.

**Box 1.** European Society of Cardiology heart failure categories by ejection fraction^3^

Category of heart failure	Abbreviation	Left ventricular ejection fraction, %	Additional criteria to meet diagnosis
Heart failure with reduced ejection fraction	HFrEF	<40	Not required
Heart failure with mid-range ejection fraction	HFmrEF	40–49	Elevated levels of natriuretic peptides and either relevant structural heart disease or diastolic dysfunction
Heart failure with preserved ejection fraction	HFpEF	≥50	Elevated levels of natriuretic peptides and either relevant structural heart disease or diastolic dysfunction

Studies that have analysed HFpEF and HFrEF separately find a difference in medication prescribed, with patients with HFrEF more likely to be treated with angiotensin converting enzyme inhibitors (ACEI), angiotensin receptor blockers (ARBs), beta-blockers (BBs) or mineralocorticoid receptor antagonists (MRAs).^17^ These treatments are effective in HFrEF but do not appear to improve survival rates in HFpEF or HFmrEF, where no treatment has thus far been convincingly shown to improve prognosis.^3^

There is an association between HFpEF and female sex, increasing age, higher systolic blood pressures, and hypertensive or valvular HF aetiology, all factors suggesting it will becoming increasingly prevalent in coming years.^16,17^ Various causes for HFpEF have been suggested, including hypertension and diabetes, which may contribute to diastolic dysfunction, ventricular stiffening, and chronotropic incompetence. HFmrEF is a relatively new concept and categorisation and treatment remain unclear.^3^

Prognosis and symptom burden appears worse with HFrEF, although different outcomes were found between studies. The Organized Program To Initiate Lifesaving Treatment In Hospitalized Patients With HF (OPTIMIZE-HF) study found that mortality during admission was lower in HFpEF (2.9% versus 3.9% in HFrEF; *P*<0.05) but there was no difference in longer-term mortality.^17^ The Acute Heart Failure Database (AHEAD) study found an increased survival rate at 1 year in participants with HFpEF of 87.0% (95% confidence interval [CI] = 84.1 to 89.7%) versus 79.4% for those with HFrEF (95% CI = 77.5to 81.3%) and also at 3 years: 73.4% (95% CI = 69.2 to 77.6%) versus 63.7% (95% CI = 61.0 to 66.4%).^16^ Patients with HFpEF may not have access to the same services such as specialist HF nurses and often have multiple comorbidities so, although their prognosis appears better, they are a cohort where significant primary care input is often needed.

## Other negative prognostic indicators

Increasing age is the factor most strongly associated with poor prognosis.^5,6,15^ Other independently associated factors related to poor prognosis reflect haemodynamic or electrolyte instability and vascular risk factors. They include: low systolic blood pressure on admission; tachycardia; high naturietic peptides (B type naturietic peptide [BNP] and N-Terminal-proBNP [NT-proBNP]); hyponatreamia; anaemia; acute kidney injury; chronic kidney disease; prior stroke; smoking; male sex; previous myocardial infarction (MI); chronic obstructive pulmonary disease; peripheral vascular disease; or significantly high or low body mass index. Symptoms are also important signs of deterioration. The New York Heart Association (NYHA) classification is used to quantify the degree of breathlessness in patients with HF as shown in Box 2.

**Box 2.** New York Heart Association classification

NYHA Class	Definition
I	No limitations of physical activity. Ordinary physical activity does not cause undue fatigue, palpitation, or dyspnoea
II	Slight limitation of physical activity. Patients are comfortable at rest. Ordinary physical activity results in fatigue, palpitation, breathlessness, or angina pectoris
III	Marked limitation of physical activity. Although patients are comfortable at rest, less than ordinary activity will lead to symptoms
IV	Inability to carry out any physical activity without discomfort. Symptoms of congestive cardiac failure are present even at rest. Increased discomfort with any physical activity

Data from the AHEAD registry includes prospectively collected information on 4153 patients hospitalised with acute HF and confirmed LVSD across seven centres in the Czech Republic. A univariate Cox proportional hazards model was used to determine predictors of long-term mortality. The five factors on admission with the highest hazard ratios for 1-year mortality were: NYHA score of III–IV; BNP >2000 pg/ml or NT-proBNP <10 000 pg/ml; diuretic use; creatinine >145 uµmol/l or creatinine clearance <40 ml/min; and age >70 years.^16^

## Prescribing in HF

**Figure 1.**
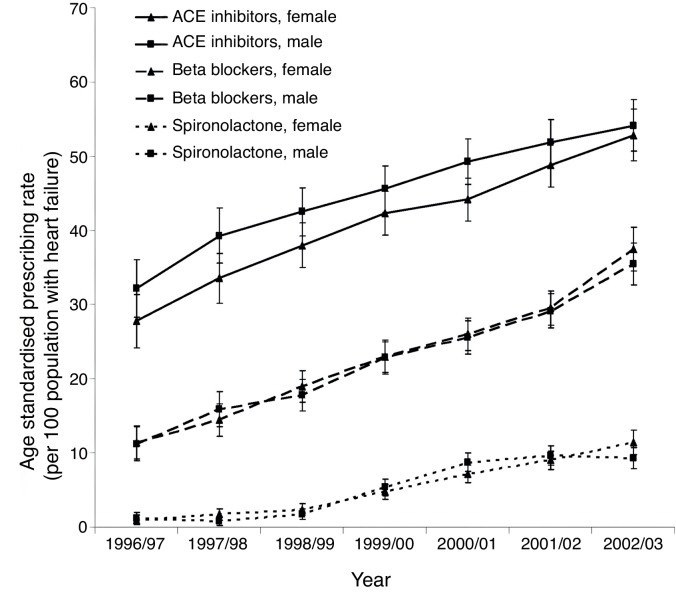
Changes in age-standardised prescribing rates between 1996 & 2003.^15^ ACE = angiotensin converting enzyme.

Reproduced with permission of Circulation & Wolters Kluwer Health, Inc.

Figure 1 shows an increase in the proportion of patients treated with cardioprotective medication, such as ACEIs, BBs, and spironolactone, in Scotland between 1986 and 2003.^15^ This reflects the growing body of evidence which, at that time, found a reduction in mortality and morbidity with ACEIs,^18^ BBs,^19^ and MRAs^20^ in patients with HFrEF. There remains, however, significant numbers of patients with HFrEF who are not on the recommended combinations of cardiovascular medication.

The most recent British Society for Heart Failure National Heart Failure Audit shows that rates of prescribing for disease-modifying medications are improving. Following hospital discharge and with specialist input 84% of patients with HFrEF were on either an ACEI or ARB, 86% on BBs, and 52% on MRAs.^21^ However, only 42% took all three. On general medical wards, 80% of patients with HFrEF were prescribed an ACEI or ARB, 80% a BB, and just 20% on both plus a MRA.^21^ The Meta-analysis Global Group in Chronic Heart Failure (MAGGIC) included data on 39 372 patients with HF from several large international cohort studies; 68.7% of patients were on either an ACEI or ARB and 34.0% were on a BB.^22^ Evidence also suggests those on treatment are frequently on suboptimal doses.^23^ There may be several reasons to explain the low prescribing rates, including difficulties in prescribing in renal failure and hypotension. However, increasing the proportion of patients on the correct combination of medication and at the optimal doses remains a key area to target for improvement.

## Causes of death

Large cohort studies recruiting participants with chronic HF show cardiovascular events are the most frequent cause of death, estimated to account for between 48.4% and 72.0% of all deaths, with 40.5% of these directly related to HF.^8,10,24^ The Rotterdam study found participants with HF were at an increased risk of cardiac events, cardiac death, and sudden cardiac death compared to those with no HF. The study reported a hazard ratio for cardiac death in participants with HF of 8.8 (95% CI = 5.9 to 13.2) and for sudden cardiac death of 10.8 (95% CI = 6.0 to 19).^5^ Cardiac causes of death, other than HF, include arrhythmias (9.9%)^24^ and MI (between 3.6 and 22.6% of deaths, with higher rates in patients with HFrEF).^10^

Respiratory disease (21.2%) and cancer (13.0%) were the most common causes for non-cardiovascular deaths in patients with chronic HF, where this has been reported.^10^ Therefore, while HF carries a significant risk of cardiovascular mortality, patients still die from other conditions in many cases. It is therefore important to consider multimorbidity and maintain a low index of suspicion for investigating and treating alternative causes for symptoms such as pneumonia or lung cancer in a breathless patient. The presence of several long-term conditions can make end-of-life care particularly challenging.

## Mortality differences over time

The prognosis for chronic HF has improved, when compared with very early studies such as the Framingham Heart and Offspring Studies (1971), where 1-year survival was 60.5% and 5-year survival was 31.5%.^25^ However, more recently, there has been no change or very modest improvements in survival.^8^ A large Scottish cohort study found an increased median survival time from 1.3 years to 2.3 years between 1986 and 2002^15^ and similarly a more recent Canadian cohort study found a minimal change in 1-year survival between 1997 and 2007, from 64.3% to 66.2% in acute HF and from 82.3% to 83.8% in chronic HF.^26^

The lack of improvement in survival time is disappointing given the number of evidence-based treatment options that have been in use over the past 20 years.^18–20^ In part this may be explained by the fact no treatment has been shown to improve survival in HFpEF or HFmrEF.^3^ Analysis of prescribing patterns does however suggest there is suboptimal use of proven treatments for patients with HFrEF,^15^ highlighting an important area of focus for GPs. Patients with HF also frequently have several comorbidities, which will impact on their prognosis, as seen by the range of mortality causes. A holistic approach to care is needed in these patients that goes beyond a simple algorithm-based HF treatment strategy.

## Discussing prognosis and HF trajectory

The Heart Failure Society of America (HFSA) suggest end-of-life planning should be done in coordination with primary care physicians and early enough to allow patients to meaningfully participate.^27^ Currently many patients with HF feel they never discuss their prognosis or possible causes of mortality with any healthcare professional, although these discussions are clearly important to help guide treatment and allow for patient-centred advanced care planning.^2^

GPs may be wary of crushing patients’ hope and so describe HF as an illness to treat rather than an incurable condition and this means the focus of primary care consultations is often on disease management alone.^2^ The pattern of illness in HF contributes to this, as doctors find it difficult to predict acute episodes of deterioration during periods of clinical stability and articulating the risk of sudden death is a further challenge.^2^ There may be an assumption that other members of the healthcare team such as a specialist nurse or cardiology consultant should take the lead in these discussions. Lack of advanced planning means patients do not get access to all available support including palliative care, which remains underutilised by patients with HF compared with other conditions such as cancer.^3,28^

## Conclusion

At a population level, epidemiological evidence shows mean survival rates in chronic HF of 80–90%, 50–60%, and 30% at 1, 5, and 10 years respectively. However, the complexity of a patient’s disease and their comorbidities makes it difficult to generalise these population studies to individuals. It is important to assess each patient individually but this evidence base may provide a useful guide for treatment and prognosis discussions.

Prognosis appears to have plateaued over time, highlighting the need for optimisation of evidence-based treatment with dose adjustment including up-titration of disease-modifying medication where appropriate. Further research is required to discover effective treatment for HFpEF and HFmrEF, but currently the focus for GPs should be on care of associated comorbidities and symptom control.^3^ The 30 days following discharge from an acute admission is a high-risk period so resources and early follow-up should be focused on these patients.

Increasing age, LVSD, significantly elevated natriuretic peptides, and other markers of vascular or renal disease are the best predictors of poor prognosis (although the evidence for peptides is limited to hospital out-patient follow-up). Local guidelines for suspected HF investigation differ across the UK and therefore GP access to community echocardiography and BNP measurement remains variable and incomplete. However, prognostic information should be used in primary care to identify patients at risk, escalate treatment where appropriate, but also hold timely conversations regarding realistic treatment expectations and consider the early need for discussions around resuscitation, end-of-life care, and involvement of palliative care services.*﻿﻿*
